# Supplementary material for: Growth and Maturity Status of Female Soccer Players: A Narrative Review
Source: Int J Environ Res Public Health. 2021 Feb 4;18(4):1448. doi: 10.3390/ijerph18041448 (PMC7913875; doi:10.3390/ijerph18041448)
Supplement: Supplementary file 1 [file ijerph-18-01448-s001.zip › 10_Supplementary Materials edit.docx]

**Supplementary Materials**

Table S1. Studies reporting ages, heights and weights of female youth soccer players by year within three intervals: 1992–2009, 2010–2017 and 2018–2020.

Table S2. Sources used in the compilation of heights and weights of adult female soccer players.

Figure S1A. Mean heights reported in studies of female youth soccer players within three intervals, 1992–2009, 2010–2017 and 2018–2020, plotted by age relative to medians and 25^th^ and 75^th^ percentiles of reference data for U.S. girls (see text for details).

Figure S1B. Mean weights reported in studies of female youth soccer players within three intervals, 1992–2009, 2010–2017 and 2018–2020, plotted by age relative to medians and 25^th^ and 75^th^ percentiles of reference data for U.S. girls (see text for details).
