# Supplementary material for: Growth and Maturity Status of Female Soccer Players: A Narrative Review
Source: Int J Environ Res Public Health. 2021 Feb 4;18(4):1448. doi: 10.3390/ijerph18041448 (PMC7913875; doi:10.3390/ijerph18041448)
Supplement: Supplementary file 1 [file ijerph-18-01448-s001.zip › 11_Table S1 edit.docx]

**Supplementary Table S1**. Studies reporting ages, heights and weights of female youth soccer players by year within three intervals: 1992–2009, 2010–2017 and 2018–2020. Studies were initially sorted by those for which raw data were available to the first author. Then, all studies considered in the narrative review were sorted within each of the three time intervals by those which reported only age, height and weight and those which also included an indicator of maturity status: skeletal age, age at menarche, pubertal status, percentage of predicted adult height and predicted maturity offset or age at peak height velocity (PHV).

**Studies for which the raw data for age, height and weight were available to the first author**

**1995–2020** (the references are also included in the three time intervals considered below)

1. Siegel SR (1995) Growth and maturity status of female soccer players from late childhood through early adulthood. Master of Science thesis, University of Texas, Austin, TX, USA. –also includes status quo and retrospective data for menarche which were re-analyzed
   1. Siegel SR, Katzmarzyk PT, Malina RM (1996) Somatotypes of female soccer players 10-24 years of age. In Studies in Human Biology, Bodzsár BE, Susanne C, (eds). Budapest, Hungary: Eötvös University Press, pp 277-85.
2. Kontos AP (2000) The effects of perceived risk, risk-taking behaviors and body size on injury in youth sport. Doctoral dissertation, Michigan State University, East Lansing, MI, USA.
   1. Kontos AP (2004) Perceived risk, risk taking, estimation of ability and injury among adolescent sport participants. J Pediatr Psychol. 29:447-55.
3. Cumming SP (2002) A bio-psychosocial investigation of self-determined motivation in recreational and travel youth soccer programs. Doctoral dissertation, Michigan State University, East Lansing, MI, USA.
   1. Cumming SP, Battista RA, Standage M, et al. (2006) Estimated maturity status and perceptions of adult autonomy support in youth soccer players. J Sports Sci. 24:1039-46. - percentage of predicted adult height attained at the time of observation
4. Stanforth PR, Crim BN, Stanforth D, et al. (2014) Body composition changes among female NCAA division I athletes across the competitive season and over a multiyear time frame. J Strength Cond Res. 28:300-7. – used only data for players 18 years of age
5. US Soccer (2018), data for age, height and weight for participants in a 2018 tournament, with permission.
6. US Soccer (2020), data for age, height and weight for participants in a 2020 tournament, with permission.

**1992–2009**

**Studies reporting only age, height and weight**

1. McCulloch RG, Bailey DA, Whalen RL, et al. (1992) Bone density and bone mineral content of adolescent soccer athletes and competitive swimmers. Ped Exerc Sci. 4:319-30.
2. Kontos AP (2000) The effects of perceived risk, risk-taking behaviors and body size on injury in youth sport. Doctoral dissertation, Michigan State University, East Lansing, MI, USA.
   1. Kontos AP (2004) Perceived risk, risk taking, estimation of ability and injury among adolescent sport participants. J Pediatr Psychol. 29:447-55.
3. Siegler J, Gaskill S, Ruby B (2003) Changes evaluated in soccer-specific power and endurance either with or without a 10-week in-season, intermittent, high-intensity training protocol. J Strength Cond Res. 17:379-87.
4. Cumming SP, Eisenmann JC, Smoll FL, et al. (2005) Body size perceptions of coaching behaviors of adolescent female athletes. Psychol Sport Exerc. 6:693-705.
5. Emery CA, Meeuwisse WH, Hartmann SE (2005) Evaluation of risk factors for injury in adolescent soccer: Implementation and validation of an injury surveillance system. Am J Sports Med. 33:1882-91.
6. Gómez López M, Moro MB (2005) Características fisiológicas de jugadoras españolas de fútbol femenino. KRONOS: Rendimiento en el Deporte 4(7):27-32.
7. Bellew JW, Gehrig L (2006) A comparison of bone mineral density in adolescent female swimmers, soccer players, and weight lifters. Pediat Phys Ther. 18:19-22.
8. Dickau L (2006) Examination of aerobic and anaerobic contributions to yo-yo intermittent recovery level 1 test performance in female adolescent soccer players. Master of Science thesis, University of Victoria, British Columbia, Canada.
9. Grandstrand SL, Pfeiffer RP, Sabick MB, et al. (2006) The effects of a commercially available warm-up program on landing mechanics in female youth soccer players. J Strength Cond Res. 20:331-5.
10. Pollard CD, Sigward SM, Ota S, et al. (2006) The influence of in-season injury prevention training on lower-extremity kinematics during landing in female soccer players. Clin J Sports Med. 16:223-7.
11. Landry SC, McKean KA, Hubley-Kozey CL, et al. (2007) Neuromuscular and lower limb biomechanical differences exist between male and female elite adolescent soccer players during an unanticipated side-cut maneuver. Am J Sports Med. 35:1888-1900.
12. Sporiš G, Čanaki M, Barišić V (2007) Morphological differences of elite Croatian female soccer players according to team position. Croatian Sports Medicine Journal/Hrvatski Športskomedicinski Vjesnik. 22:91-6.
13. Almagià Flores AA, Rodríguez Rodríguez F, Barraza Gómez FO, et al. (2008) Perfil antropométrico de jugadoras Chilenas de fútbol femenino. Internat J Morphol. 26:817-21.
14. Steffen K, Myklebust G, Andersen TE, et al. (2008) Self-reported injury history and lower limb function as risk factors for injuries in female youth soccer. Am J Sports Med. 36:700-8.
15. Sigward SM, Ota S, Powers CM (2008) Predictors of frontal plane knee excursion during a drop land in young female soccer players. J Orthop Sports Phys Ther. 38:661-7.
16. Vescovi JD, McGuigan MR (2008) Relationships between sprinting, agility, and jump ability in female athletes. J Sports Sci. 26:97-107.
17. Barber Foss KD, Ford KR, Myer GD, Hewett TE (2009) Generalized joint laxity associated with increased medial foot loading in female atheltes. J Athl Train. 44:356-62.
18. Barbero-Álvarez JC, Barbero Álvarez V, Gómez M, et al. (2009) Análisis cinemático del perfil de actividad en jugadoras infantiles de fútbol mediante tecnologia GPS. KRONOS: Rendimiento en el Deporte 8(14):35-42.
19. DiStefano LJ, Padua DA, DiStefano MJ, Marshall SW (2009) Influence of age, sex, technique, and exercise program on movement patterns after an anterior cruciate ligament injury prevention program in youth soccer players. Am J Sports Med. 37:495-505.
20. Ekegran CL, Miller WC, Celebrini RG, et al. (2009) Reliability and validity of observational risk screening in evaluating dynamic knee valgus. J Orthop Sports Phys Ther. 39:665-74.
21. Mujika I, Santisteban J, Impellizzeri FM, et al. (2009) Fitness determinants of success in men’s and women’s football. J Sports Sci. 27:107-14.

**Studies including a maturity indicator in addition to age, height and weight: age at menarche**

1. Siegel SR (1995) Growth and maturity status of female soccer players from late childhood through early adulthood. Master of Science thesis, University of Texas, Austin, TX, USA. –also includes status quo and retrospective data for menarche
   1. Siegel SR, Katzmarzyk PT, Malina RM (1996) Somatotypes of female soccer players 10-24 years of age. In Studies in Human Biology, Bodzsár BE, Susanne C, (eds). Budapest, Hungary: Eötvös University Press, pp 277-85.
2. Düppe H, Gärdsell P, Johnell O, et al. (1996) Bone mineral density in female junior, senior and former football players. Osteopor Internat. 6:437-41.
3. Casper RC, Michaels J, Simon K (1997) Body perception and emotional health in athletes: A study of female adolescents involved in aesthetic sports. In Simopoulos AP, Pavlou KN (eds) Nutrition and Fitness: Metabolic and Behavioral Aspects in Health and Disease. Basel, Karger, World Review of Nutrition and Dietetics 82:134-47.
4. Pettersson U, Nordström P, Alfredson H, et al. (2000) Effect of high impact activity on bone mass and size in adolescent females: A comparative study between two different types of sports. Calc Tissue Internat. 67:207-14.
5. Södermann K, Bergström E, Lorentzon R, et al. (2000) Bone mass and muscle strength in young female soccer players. Calc Tissue Internat. 67:297-303.

**Studies including a maturity indicator in addition to age, height and weight: percentage of predicted adult height attained at the time of observation**

1. Cumming SP (2002) A bio-psychosocial investigation of self-determined motivation in recreational and travel youth soccer programs. Doctoral dissertation, Michigan State University, East Lansing, MI, USA.
   1. Cumming SP, Battista RA, Standage M, et al. (2006) Estimated maturity status and perceptions of adult autonomy support in youth soccer players. J Sports Sci. 24:1039-46.

**2010–2017**

**Studies reporting only age, height and weight**

1. Bunc V (2010) Physiological and functional characteristics of adolescent athletes in several sports. In Coelho-e-Silva MJ, Figueiredo AJ, Elferink-Gemser M, Malina, RM (editors), Youth Sports: Growth, Maturation and Talent. Coimbra, Portugal, University of Coimbra Press, p. 115-25.
2. Castagna C, Impellizzeri FM, Manzi V, et al. (2010) The assessment of maximal aerobic power with the multistage fitness test in young women soccer players. J Strength Cond Res. 24:1488-94.
3. Ortiz A, Trudelle-Jackson E, McConnell K, et al. (2010) Effectiveness of a 6-week injury prevention program on kinematics and kinetic variables in adolescent female soccer players: A pilot study. Puerto Rico Health Sci. J 29:40-8.
4. Vescovi JD, Van Heest JL (2010) Effects of an anterior cruciate ligament injury prevention program on performance in adolescent female soccer players. Scand J Med Sci Sports. 20:394-402.
5. Ferry B, Duclos M, Burt L, et al. (2011) Bone geometry and strength adaptations to physical constraints inherent in different sports: Comparison between elite female soccer players and swimmers. J Bone Min Metab. 29:342-51.
   1. Ferry B, Lespessailles E, Rochcongar P, et al. (2013) Bone health during late adolescence: Effects of an 8-month training program on bone geometry in female athletes. J Bone Spine. 80:57-63. – same age, height, weight data as 2011 study
6. Gibson JC, Stuart-Hill L, Martin S, et al. (2011) Nutrition status of junior elite Canadian female soccer athletes. Internat J Sport Nutr Exerc Metab. 21:507-14.
7. Kontos AP, Dolese A, Elbin III RJ, et al. (2011) Relationship of soccer heading to computerized neurocognitive performance and symptoms among female and male youth soccer players. Brain Inj. 25:1234-41.
8. Parsons JL, Carswell J, Nwboa IM, et al. (2011) Athletic perceptions and physical performance effects of the FIFA 11+ program in 9-11 year-old female soccer players: A cluster randomized trial. Int J Sports Phys Ther. 14:740-52.
9. Rowland T, Unnithan V, Roche D, et al. (2011) Myocardial function and aerobic fitness in adolescent females. Eur J Appl Physiol. 111:1991-7.
10. Rubley MD, Haase AC, Holcomb WR, et al. (2011) The effect of plyometric training on power and kicking distance in female adolescent soccer players. J Strength Cond Res. 25:129-34.
11. Stieg JL, Faulkinbury KJ, Tran TT, et al. (2011) Acute effects of depth jump volume on vertical jump performance in collegiate women soccer players. Kinesiology 43:25-30.
12. Celebrini RG, Eng JJ, Miller WC, et al. (2012) The effect of a novel movement strategy in decreasing ACL risk factors in female adolescent soccer players. J Strength Cond Res. 26:3404-17.
13. Chrisman SP, O’Kane JW, Polissar NL, et al. (2012) Strength and jump biomechanics of elite and recreational female youth soccer players. J Athl Train. 47:609-15.
14. Dillern T, Ingebrigtsen J, Shalfawi SAI (2012) Aerobic capacity and anthropometric characteristics of elite-recruit female soccer players. Serb J Sports Sci 6:43-9.
15. Lindblom H, Waldén M, Hägglund M (2012) No effect on performance tests from a neuromuscular warm-up programme in youth female football: A randomized controlled trial. Knee Surg Sports Traumatol Arthrosc. 20:2116-23.
16. Portela Sarazola J (2012) Description and exemplary analysis of match running performance and a selected battery of tests in adolescent female soccer players. Master’s thesis, Institute of Movement and Training Science, University of Leipzig, Germany (as cited by Martinez-Lagunas et al. [J Sport Health Sci. 2014; 3:258-72]).
17. Walden M, Atroshi I, Magnusson H, et al. (2012) Prevention of acute knee injuries in adolescent female football players: Cluster randomized control trial. Br Med J. 344:e3042, doi: 10.1136/bmj.e3042.
18. Castagna C, Castellini E (2013) Vertical jump performance in Italian male and female national team soccer players. J Strength Cond Res. 27:1156-61.
19. Guagliano JM, Rosenkranz RR, Kolt GS (2013) Girls’ physical activity levels during organized sports in Australia. Med Sci Sports Exerc. 45:116-22.
20. Noyes FR, Barber-Westin SD, Tutalo Smith ST, et al. (2013) A training program to improve neuromuscular and performance indices in female high school soccer players. J Strength Cond Res. 27:240-51.
21. Adhikari A, Nugent J (2014) Anthropometric characteristic, body composition and somatotype of Canadian female soccer players. Am J Sports Sci. 2:14-8.
22. Celebrini RG, Eng JJ, Miller WC, et al. (2014) Effect of a novel movement strategy in decreasing ACL risk factors in female adolescent soccer players: A randomized controlled trial. Clin J Sports Med. 24: 134-41.
23. Gutierrez GM, Conte C, Lightbourne K (2014) The relationship between impact force, neck strength and neurocognitive performance in soccer heading in adolescent females. Pediat Exerc Sci. 26:33-40.
24. Haugen TA, Tønnessen E, Hem E, et al. (2014) VO2max characteristics of elite female soccer players 1989-2007. Internat J Sports Physiol Perf. 9:515-21.
25. Manson SA, Brughelli M, Harris NK (2014) Physiological characteristics of international female soccer players. J Strength Cond Res. 28:308-18.
26. Mathisen GE, Danielsen KH (2014) Effects of speed exercises on acceleration and agility performance in 13-year-old female soccer players. J Phys Educ Sport. 14:471-4.
27. Rumpf MC, Schneider AS, Schneider C, Mayer HM (2014) Training profiles and motivation of male and female youth soccer players. Int J Sports Sci Coach. 9:207-16.
28. Sogabe N, Okada M, Tokita Y, et al. (2014) Physical status and eating habits of junior/senior high school girls playing football. J Jpn Soc Shokuiku (nutrition education). 8:41-47. (in Japanese)
29. Stanforth PR, Crim BN, Stanforth D, et al. (2014) Body composition changes among female NCAA division I athletes across the competitive season and over a multiyear time frame. J Strength Cond Res. 28:300-7. – used only data for soccer players 18 years of age
30. Hirose N, Nakahori C (2015) Age differences in change-of-direction performance and its sub-elements in female football players. Internat J Sports Physiol Perf. 10:440-5.
31. Inoue Y, Otania Y, Naruse S, et al. (2015a) Relationships between lower-extremity muscle strength and the modified star excursion balance test in adolescent female players. Rigakuryoho Kagaku. 30:853-6 (in Japanese).
32. Inoue Y, Otani Y, Eusugi M, et al. (2015b) Characteristics of injuries with female junior high and high school soccer players in comparison with male players. Jpn J Phys Fit Sports Med. 64:345-50 (in Japanese).
33. Mathisen GE, Pettersen SA (2015) The effect of speed training on sprint and agility performance in 15-year-old female soccer players. LASE J Sport Sci. 6:61-70.
34. Nevado-Garrosa F, Suarez-Arrones L (2015) Comparación de las demandas fisicas de tareas de fútbol reducido y la competición en jugodoras de fútbol sub 13. Cultura, Ciencia y Deporte (Universidad Católica de Murcia) 10:235-43.
35. Ono E, Maegawa T, Kamei Y, et al. (2015) Investigating the method of evaluating the ability of power exertion at the lower limb for female collegiate athletes. Japan J Coaching Stud. 28:175-82.
36. Datson N (2016) An analysis of the physical demands of international female soccer match-play and the physical characteristics of elite players. Doctoral dissertation, Liverpool John Moores University, Liverpool, United Kingdom.
37. Hägglund M, Waldén M (2016) Risk factors for acute knee injury in female youth football. Knee Surg Sports Traumatol Arthrosc. 24:737-46.
38. Lesinski M, Muehlbauer T, Granacher U (2016) Concurrent validity of the Gyko inertial sensor system for the assessment of vertical jump height in female sub-elite youth soccer players. BMC Sports Sci Med Rehab. 8:35, 1-9, doi: 10.1186/s13102-016-0061-x.
39. Oyón P, Franco L, Rubio FJ, Valero A (2016) Young women soccer players: Anthropmetric and physiological characteristics – evolution in a sports season. Archivos de Medicina del Deporte 33:24-8.
40. Póvoas SCA, Castagna C, da Costa Soares JM, et al. (2016) Reliability and construct validity of yo-yo tests in untrained and soccer-trained schoolgirls aged 9-16. Pediat Exerc Sci. 28:321-30.
41. dos Santos Andrade M, Mascarin NC, Benedito-Silva AA, et al. (2016) Assessment of isokinetic peak torque reliability of the hip flexor, extensor, adductors and abductors muscles in female soccer players from 14 to 25 years. J Sports Med Phys Fit. 56:843-8.
42. Turner E (2016) Physical and match performance of female soccer players. Doctoral dissertation, University of Salford, Manchester, United Kingdom.
43. Chapelle L, Tassignon B, Aerenhouts D, et al. (2017) The hydration status of young female elite soccer players during an official tournament. J Sports Med Phys Fit. 57:1186-94.
44. Gradidge PJ-L, Constantinou D (2017) A comparative study on the cardiac morphology and vertical jump height of adolescent Black South African male and female amateur competitive footballers. Cardiovasc J Africa. 28: online publication. Doi: 10:5830/CVJA-2017-032.
45. Manore MM, Patton-Lopez MM, Meng Y, et al. (2017) Sport nutrition knowledge, behaviors and beliefs of high school soccer players. Nutrients 9:350, doi:10.3390/nu9040350.
46. Nguyen A-D, Zuk EF, Baellow AL, et al. (2017) Longitudinal changes in hip strength and range of motion in female youth soccer players: Implications for ACL injury – a pilot study. J Sport Rehab. 26:358-64.
47. Richardson A, Clarsen B, Verhagen EALM, Stubbe JH (2017) High prevalence of self-reported injuries and illnesses in talented female athletes. BMJ Open Sport Exerc Med. 3:e000199, doi:10.1136/bmjsem-2016-000199.
48. Taylor JB, Ford KR, Schmitz RJ, et al. (2017) Biomechanical differences of multidirectional jump landings among female basketball and soccer players. J Strength Cond Res. 31:3034-45.
49. Thompson JA, Tran AA, Gatewood CT, et al. (2017) Biomechanical effects of an injury prevention program in preadolescent female soccer athletes. Am J Sports Med. 45:294-301.
50. Watson A, Brickson S, Brooks A, et al. (2017) Subjective well-being and training load predict in-season injury and illness risk in female youth soccer players. Br J Sports Med. 51:194-99.

**Studies including a maturity indicator in addition to age, height and weight: age at menarche**

1. Brännström A, Yu J-G, Jonsson P, et al. (2017) Vitamin D in relation to bone health and muscle function in young female soccer players. Eur J Sport Sci. 17:249-56.

**Studies reporting only age at menarche**

1. Prather H, Hunt D, McKeon K, et al. (2016) Are elite female soccer athletes at risk for disordered eating attitudes, menstrual dysfunction, and stress fractures? Phys Med Rehab 8:208-13. – heights and weights of players were not reported

**Studies including a maturity indicator in addition to age, height and weight: pubertal status**

1. Anauate Nicolao AL, Pedrinelli A, Martino Zogalb PA, et al. (2010) Influência da maturação sexual no limiar de lactate em jogadoras de futebol. Revista Brasileira de Medicina do Esporte 16:335-38.
2. Lyle MA, Sigward SM, Tsai L-C, et al. (2011) Influence of maturation on instep kick biomechanics in female soccer athletes. Med Sci Sports Exerc. 43:1948-54.
3. Sigward SM, Pollard CD, Havens KL, Powers CM (2012) Influence of sex and maturation on knee mechanics during side-step cutting. Med Sci Sports Exerc. 44:1497-1503.
   1. Sigward SM, Pollard CD, Powers CM (2012) The influence of sex and maturation on landing biomechanics: implications for anterior cruciate ligament injury. Scand J Med Sci Sport 22:502-9. – same data set
4. Ubago-Guisado E, Gómez-Cabello A, Sánchez-Sánchez J, et al. (2015) Influence of different sports on bone mass in growing girls. J Sports Sci. 33:1710-8.
   1. Ubago-Guisado E, Mata E, Sánchez-Sánchez J, et al. (2017) Influence of different sports on fat mass and lean mass in growing girls. J Sport Health Sci. 6:213-8. – same data set
   2. Ubago-Guisado E, Garcia-Unanue J, López-Fernández J, et al. (2017) Association of different types of playing surfaces with bone mass in growing girls. J Sports Sci. 35:1484-92. – same data set
5. Unnithan V, Roche DM, Garrard M, et al. (2015) Oxygen uptake kinetics in trained adolescent females. Eur J Appl Physiol. 115:213-20.
6. Plaza-Carmona M, Vicente-Rodríguez G, Gómez-Cabello A, et al. (2016) Higher bone mass in prepubertal and peripubertal female footballers. Eur J Sport Sci. 16:877-83
   1. Plaza-Carmona M, Ubago Guisado E, Sánchez-Sánchez J, et al. (2013) Body composition and physical fitness in prepubertal girls swimmers and soccer players. J Sport Hlth Res. 5:251-8. – same data set
7. Lozano Berges G, Matute Llorente A, Gómez Bruton A, et al. (2017) Body fat percentage comparisons between four methods in young football players: Are they comparable? Nutrición Hospitalaria 34:1119-24.

**Studies including a maturity indicator in addition to age, height and weight: predicted maturity offset or age at PHV**

1. Taylor JM, Portas MD, Wright MD, et al. (2012) Within-season variation of fitness in elite youth female soccer players. J Athletic Enhance. 1:2; doi.org/10.4172/2324-9080.1000102.
2. de Ste Croix MBA, Priestley AM, Lloyd RS, et al. (2015) ACL injury risk in elite female youth soccer: Changes in neuromuscular control of the knee following soccer-specific fatigure. Scan J Med Sci Sports. 25:e531-38.
   1. de Ste Croix M, Priestley A, Lloyd R, et al. (2018) Age-related differences in functional hamstring/quadriceps ratio following soccer exercise in female youth players: An injury risk factor. Pediat Exerc Sci. 30:376-82. – same age, height, weight data as 2015 study
3. Wright MD, Hurst C, Taylor JM (2016) Contrasting effects of a mixed-methods high-intensity interval training intervention in girl football players. J Sports Sci. 34:1808-15.
4. Emmonds S, Morris R, Murray E, et al. (2017) The influence of age and maturity status on the maximum and explosive strength characteristics of elite youth female soccer players. Sci Med Football. 1:209-15. (mean CAs from reference a below)
   1. Emmonds S, Till K, Redgrave J, et al. (2018) Influence of age on the anthropometric and performance characteristics of high-level youth female soccer players. Int J Sports Sci Coach. 13:779-86. – descriptive stats for CA, HT, WT for U10-U12-U14-U16
   2. Emmonds S, Sawczuk T, Scantlebury S, et al. (2018) Seasonal changes in the physical performance of elite youth female soccer players. J Strength Cond Res. 24 October, doi:10.1519/JSC.0000000000002943, online ahead of print.
   3. Emmonds S, Scantlebury S, Murray E, et al. (2018) Physical characteristics of elite youth female soccer players characterized by maturity status. J Strength Cond Res. 7 September, doi: 10.1519/JSC.0000000000002795, online ahead of print.
5. Lesinski M, Prieske O, Helm N, et al. (2017) Effects of soccer training on anthropometry, body composition, and physical fitness during a soccer season in female elite young athletes: A prospective cohort study. Front Physiol. 8:1093, doi:10.3389/fphys.2017.01093. – maturity offset … “Biological age (i.e., pre-PHV, PHV, post-PHV) was determined according to Mirwald et al. (2002) using time from PHV, based on the Tanner 5-point scale (Marshall and Tanner, 1959)” (p 3).

**2018–2020**

**Studies reporting only age, height and weight**

1. Bishop C, Read P, Mc Cubbine J, et al. (2018) Vertical and horizontal asymmetries are related to slower sprinting and jump performance in elite youth female soccer players. J Strength Cond Res. doi.org/10.1519/JSC.0000000000002544, online ahead of print.
2. Braun H, von Andrian-Werburg J, Schänzer W, et al. (2018) Nutrition status of young elite female German football players. Pediat Exerc Sci. 30:159-69.
3. Cabistany D, Pinheiro E, Rico AM, et al. (2018) Características neuromusculares de jogadoras de futebol: Estudo com jovens atletas de Pelotas-RS. Revista Brasileira de Futsal e Futebol 10: 11-7.
4. Caccese JB, Buckley TA, Tierney RT, et al. (2018) Sex and age differences in head acceleration during purposeful soccer heading. Res Sports Med. 26: 64–74.
5. Chan IS, Fu L-L (2018) Unstable surface training effects on balance and lower limb power in adolescent female soccer players. Med Sci Sports Exerc. 50:S781, 3176 (abstract).
6. Cherian KS, Shahkar F, Sainoji A, et al. (2018) Resting metabolic rate of Indian junior soccer players: Testing agreement between measured versus selective prediction equations. Am J Hum Biol. 30(1):Epub23066. Doi:10:1002/ajhb.23066.
   1. Cherian KS, Sainoji A, Nagalla B, Yagnambhatt VR (2018) Energy balance coexists with disproportionate micronutrient consumption across training, during pretraining, and post-training among Indian junior soccer players. Pediatr Exerc Sci. 30:506-515. – same age, height, weight data
7. Cross KM, Gurka KK, Saliba S, et al. (2018) Comparison of thigh muscle strain occurrence and injury patterns between male and female high school soccer athletes. J Sport Rehab. 27:451–9.
8. Gutiérrez Manzanedo JV, Fernandez Santos JdR, Ponce Gónzalez JG, et al. (2018) Extensibilidad isquiosural en jugadoras de élite de fútbol. Retos (Federación Española de Asociaciones de Docentes de Educación Física [FEADEF]) 33:175-78.
9. Harriss A, Walton DM, Dickey JP (2018) Direct player observation is needed to accurately quantify heading frequency in youth soccer. Res Sports Med. 26:191-8.
10. Hughes JD, Denton K, Lloyd RS, et al. (2018) The impact of soccer match play on the muscle damage response in youth female athletes. Internat J Sports Med. 39:343-8.
11. Oddsson HR (2018) Athlete profile: Basic anthropometry, physical fitness and specific skill of the Icelandic female youth national teams: A descriptive analysis. Master of Science thesis. Reykjavik University, Iceland.
12. Patton-Lopez MM, Manore MM, Branscum A, et al. (2018) Changes in sport nutrition knowledge, attitudes/beliefs and behaviors following a two-year sport nutrition education and life-skills intervention among high school soccer players. Nutrients 10(11):1636, doi:10.3390/nu10111636.
13. Perroni F, Gallotta MC, Pisano S, et al. (2018) Gender differences in anthropometric parameters and technical performance of youth soccer players. Sport Sciences for Health, <https://doi.org/10.1007/s11332-018-0456-z>.
14. Poehling RA (2018) Monitoring explosive performances in relation to training load accumulation in adolescent female soccer players. Master of Science thesis, University of British Columbia, Vancouver, British Columbia.
15. Prieske O, Maffiuletti NA, Granacher U (2018) Postactivation potentiation of the plantar flexors does not directly translate to jump performance in female elite young soccer players. Front Physiol. 9:276, doi:10.3389/fphys.2018.00278, eCollection 2018.
16. Raisanen AM, Arkkila H, Vasankari T, et al. (2018) Investigation of knee control as a lower extremity injury risk factor: A prospective study in youth football. Scand J Med Sci Sports. 28: 2084-92.
17. Sha J-B, Zhang S-S, Lu Y-M, et al. (2018) Effects of the long-term consumption of hydrogen-rich water on the antioxidant activity and the gut flora in female juvenile soccer players from Suzhou, China. Med Gas Res. 8(4):135-43. Doi:10.4103/2045-9912.248263.
18. Sugimoto D, Howell DR, Tocci NX, et al. (2018) Risk factors associated with self-reported injury history in female youth soccer players. Physician Sportsmed. 46(3):312-8.
19. Thompson-Kolesar JA, Gatewood CT, Tran AA, et al. (2018) Age influences biomechanical changes after participation in an anterior cruciate ligament injury prevention program. Am J Sports Med. 46:598-606. – used only data for 15 years, data for11 years reported in Thompson et al. (2017)
20. US Soccer (2018), data for age, height and weight for participants in a 2018 tournament, with permission.
21. van den Tillaar R (2018) Comparison of step-by-step kinematics in repeated 30-m sprints in female soccer players. J Strength Cond Res. 32:1923-8.
22. Vargas VZ, Baptista AF, Pereira GOC, et al. (2018) Modulation of isometric quadriceps strength in soccer players with transcranial direct current stimulation: A crossover study. J Strength Cond Res. 32:1336-41.
23. Watson A, Brickson S (2018) Impaired sleep mediates the negative effects of training load on subjective well-being in female youth athletes. Sports Health 10:244-9.
24. Bishop C, Pereira LA, Reis VP, et al. (2019) Comparing the magnitude and direction of asymmetry during the squat, countermovement and drop jump tests in elite youth female soccer players. J Sports Sci. 29:1-8.
25. Chatzopoulos D, Kapodistria L, Doganis G, et al. (2019) Effects of varying volumes of dynamic stretching on active range of motion, reaction time, and movement time in female soccer players. J Exerc Physiol on line, 22(5):147-56.
26. Eustace SJ, Page M, Greig M (2019) Isokinetic strength differences between elite senior and youth female soccer players identifies training requirements. Phys Ther Sport. 39:45-51.
27. Gerling S, Loose O, Zant R, et al. (2019) Echocardiographic diagnosis of congenital coronary artery abnormalities in a continuous series of adolescent football players. Eur J Prev Cardiol. Doi: 10.1177/2047487319825520.
28. Gonzalez-de Los Reyes Y, Fernandez-Ortega J, Garavito-Peña F (2019) Características de fuerza y velocidad de ejecución en mujeres jóvenes futbolistas. Revista Internacional de Medicina y Ciencias de la Actividad Fisica y el Deporte 19:167-79.
29. González-Garcia J, Morencos E, Balsalobre-Fernández C, et al. (2019) Effect of 7-week hip thrust versus back squat resistance training on performance in adolescent female soccer players. Sports 7:80, 1-13, doi:10.3390/sports7040080.
30. Hannon JP, Wang-Price S, Garrison JC, et al. (2019) Normalized hip and knee strength in two age groups of adolescent female soccer players. J Str Cond Res. doi: 10.1519/JSC.0000000000003420
31. Harriss AB, Abbott K, Kimpinski K, et al. (2019) An evaluation of heart rate variability in female youth soccer players following soccer heading: A pilot study. Sports 7:229, 1-7, doi:10.3390/sports7110229.
32. Higinbotham SE, Wexler R, Colson R, et al. (2019) Age and knee confidence effects on LESS and LESS-RMC scores in female youth soccer players. Med Sci Sports Exerc. 51:S63, 271 (abstract).
33. Höner O, Raave J, Murr D, et al. (2019) Prognostic relevance of motor tests in elite girls’ soccer: a five-year prospective cohort study within the German talent promotion program. Sci Med Football 3:287-96.
34. Jeras NMJ, Bovend’Eerdt TJH, McCrum C (2019) Biomechanical mechanisms of jumping performance in youth elite female soccer players. J Sports Sci. doi:10.1080/02640414.2019.1674526, on line ahead of print.
35. Miller LE, Pinkerton EK, Fabian KC, et al. (2019) Characterizing head impact exposure in youth female soccer with a custom-instrumented mouthpiece. Res Sports Med. 28:55-71.
36. Pardos-Mainer E, Casajus JA, Gonzalo-Skok O (2019) Adolescent female soccer players’ soccer-specific warm-up effects on performance and inter-limb asymmetries. Biol Sport 36: 199–207.
37. Pérez S, Rodríguez A, Sánchez A, et al. (2019) Efecto de los juegos reducidos sobre jugadoras de futbol. Revista Internacional de Medicina y Ciencias de la Actividad Fisica y el Deporte 19:371-86.
38. Ramos GP, Nakamura FY, Penna EM, et al. (2019) Comparison of physical fitness and anthropometrical profiles among Brazilian female soccer national teams from U15 to senior categories. J Strength Cond Res, doi:10.1519/JSC.0000000000003140, online ahead of print.
39. Sugimoto D, Borg DR, Brilliant AN, et al. (2019) Effect of sports and growth on hamstrings and quadriceps development in young female athletes: Cross-sectional study. Sports. 7(7):158; doi:10.3390/sports7070158.
40. Watson A, Brickson S (2019) Relationships between sport specialization, sleep, and subjective well-being in female adolescent athletes. Clin J Sports Med. 29:384-90.
41. Zuk B, Sutkowski M, Pasko S, et al. (2019) Posture correctness of young female soccer players. Sci Reports. 9:11179, doi.org/10.1038/s41598-019-47619-1.
42. Adigüzel NS, Koç M (2020) Comparison of dynamic balance test scores of young female volleyball and soccer players. Int J Appl Exerc Physiol 9:100-4.
43. Andrade MS, Junqueira MS, Barbosa de Lire CA, et al. (2020) Age-related differences in torque in angle-specific and peak torque to quadriceps ratios in female soccer players from 11-10 18 years old: A cross-sectional study. Res Sports Med. doi:10.1080/15438627.2020.1742713
44. Fernandez Ortega JA, Gonzalez De los Reyes Y, Garavito Peña FR (2020) Effects of strength training based on velocity versus traditional training on muscle mass, neuromuscular activation, and indicators of maximal power and strength in girls soccer players. Apunts Sports Med. 55:53-61.
45. Grooten WJA, Karlefur O, Conradsson D (2020) Effects of verbal knee alignment instructions on knee kinematics, kinetics and the performance of a single-leg jump in female adolescent soccer players. Eur J Physiother. 22:106-14.
46. Hammami MA, Ben Klifa W, Ben Ayed K, et al. (2020) Physical performances and anthropometric characteristics of young elite North-African female soccer players compared with international standards. Sci Sports. 35:67-74.
47. Harkness-Armstrong A, Till K, Datson N, et al. (2020) Technical characteristics of elite youth female soccer match-play: Position and age group comparisons between under 14 and under 16 age groups. Int J Perf Analysis Sport. https.doi.org/10.1080/24748668.2020.1820173
48. Harriss A, Johnson AM, Thompson JWG, et al. (2020) Cumulative soccer heading amplifies the effects of brain activity observed during concurrent moderate exercise and continuous performance task in female youth soccer players. Journal of Concussion 4:1-9.
49. Lanhers C, Courteix D, Valente-dos-Santos J, et al. (2020) Gonadal hormones may predict structural bone fragility in elite female soccer player. J Sports Sci. 38:827-37.
50. Lim K-H, Seo T-B, Kim Y-P (2020) Relationship between movement dysfunctions and sports injuries according to gender of youth soccer players. J Exerc Rehab 16:427-31.
51. Millar NA, Colenso-Semple LM, Lockie RG, et al. (2020) In-season hip thrust vs. back squat training in female high school soccer players. Internat J Exer Sci. 13:49-61.
52. Pambo P, Adu-Adadey M, Ankrah PT, et al. (2020) Electrocardiographic and echocardiographic findings in Ghanaian female soccer players. Clin J Sports Med. doi:10.1097/JSM.0000000000000851, on line ahead of print.
53. Sonesson S, Lindblom H, Hägglund M (2020) Performance on sprint, agility and jump tests have moderate to strong correlations in youth football players but performance tests are weakly correlated to neuromuscular control tests. Knee Surg Sports Traumatol Arthroscopy <https://doi.org/10.1007/s00167-020-06302-z>.
54. US Soccer (2020), data for age, height and weight for participants in a 2020 tournament, with permission.
55. Vargas VZ, Motta C, Peres B, et al. (2020) Knee isokinetic muscle strength and balance ratio in female soccer players of different age groups: a cross-sectional study. Physician Sportsmed. 48(1):105-9.

**Studies including a maturity indicator in addition to age, height and weight: pubertal status**

1. Lozano-Berges G, Matute-Llorente A, Gómez-Bruton A, et al. (2019) Accurate prediction equation to assess body fat in male and female adolescent football players. Internat J Sport Nutr Exerc Metab. 29:297-302.
2. Lozano-Berges G, Matute-Llorente A, Gómez-Bruton A, et al. (2019) Is playing soccer more osteogenic for females before the pubertal spurt? J Hum Kinet. 67:153-61.

**Studies including a maturity indicator in addition to age, height and weight: predicted maturity offset or age at PHV**

1. de Ste Croix M, Hughes J, Ayala F, et al. (2018) Efficacy of injury prevention training is greater for high-risk vs low-risk elite female youth soccer players. Am J Sports Med. 46:3271-80.
2. Lozano-Berges G, Matute-Llorente A, Gómez-Bruton A, et al. (2018) Bone geometry in young male and female football players: A peripheral quantitative computed tomography (pQCT) study. Arch Osteopor. 13(1):57, [doi.org/10.1007/s11657-018-0472-2](https://doi.org/10.1007/s11657-018-0472-2).
3. Pardos-Mainer E, Casajus JA, Gonzalo-Skok O (2019) Reliability and sensitivity of jumping, linear sprinting and change of direction ability tests in adolescent female football players. Sci Med Football 3:183-90. – predicted maturity offset of players 14.2±1.6 years was -0.66±0.98 year and of 17.1±0.8 years was 0.41±0.32 year; both were not consistent with other studies (see Figure 3)
4. Wright MD, Innerd A (2019) Application and interpretation of the yo-yo intermittent recovery test to the long term physical development of girls’ association football players. Sci Med Football. 3:297-306. – mixed-longitudinal age group data over four seasons (Figure 1) – corrected means provided by MD Wright (29 June 2020)
5. Wright MD, Atkinson G (2019) Changes in sprint-related outcomes during a period of systematic training in a girls’ soccer academy. J Strength Cond Res. 33:793-800.
6. Zinke R, Gebel A, Granacher U, Prieske O (2019) Acute effects of short-term local tendon vibration on plantar flexor torque, muscle contractile properties, neuromuscular and brain activity in young athletes. J Sports Sci Med. 18:327-36.
7. Baxter-Jones ADG, Barbour-Tuck EN, Dale D, et al. (2020) The role of growth and maturation during adolescence on team-selection and short-term sports participation. Ann Hum Biol. 47:316-323.
8. Lesinski M, Prieske O, Chaabene H, et al. (2020) Seasonal effects of strength endurance vs power training in young female soccer players. J Str Cond Res. https://doi:10.1519/JSC.0000000000003564.
9. Obrien-Smith J, Bennett KJM, Fransen J, et al. (2020) Same or different? A comparison of anthropometry, physical fitness and perceptual motor characteristics in male and female youth soccer players. Sci Med Football 4:37-44.
10. Pardos-Mainer E, Casajus J, Bishop C, et al. (2020) Effects of combined strength and power training on physical performance and inter-limb asymmetries in adolescent female soccer players. Internat J Sports Physiol Perf. doi: 10.1123/ijspp.2019-0265.
11. Wright MD, Songane F, Emmonds S, et al. (2020) Differential ratings of perceived match and training exertion in youth female soccer. Int J Sports Physiol Perf. 18:1-9, doi:10.1123/ijspp.2019-0595.

**Studies including a maturity indicator in addition to age, height and weight: percentage of predicted adult height attained at the time of observation**

1. Mullen CM, Taylor JB, Aube MA, et al. (2018) Effect of maturation on heart rate during a six-week plyometric training in female soccer players. Med Sci Sports Exerc. 50:778-9 (abstract 3168).
2. Westbrook AE, Taylor JB, Nguyen A-D, et al. (2020) Effects of maturation on knee biomechanics during cutting and landing in young female soccer players. PLoS One. 15(5):e0233701, 1-11.

**Studies including a maturity indicator in addition to age, height and weight: skeletal age**

1. Martinho DV, Coelho-e-Silva MJ, Gonçalves-Santos J, et al. (2020) Height, body mass and skeletal maturity of female soccer players aged 11-16 years (under review).
   1. Martinho DV, Coelho-e-Silva MJ, Valente-dos-Santos J, et al. (2020) Assessment of biological maturation in youth female soccer players: Agreement between Greulich-Pyle and Fels protocols. Under review 2020.
