# Supplementary material for: Growth and Maturity Status of Female Soccer Players: A Narrative Review
Source: Int J Environ Res Public Health. 2021 Feb 4;18(4):1448. doi: 10.3390/ijerph18041448 (PMC7913875; doi:10.3390/ijerph18041448)
Supplement: Supplementary file 1 [file ijerph-18-01448-s001.zip › 12_Table S2 edit.docx]

**Supplementary Table** S2. Sources used in the compilation of heights and weights of adult female soccer players.

Reviews:

1. Datson N, Hulton A, Andersson H, et al. Applied physiology of female soccer: An update. Sports Med 2014; 44:1225-40
2. Davis JA, Brewer J. Applied physiology of female soccer players. Sports Med. 1993; 16:180-9.
3. Martínez-Lagunas V, Niessen M, Hartmenn U. Women’s football: Player characteristics and demands of the game. J Sport Sci Hlth 2014; 3:258-72.
4. Scott D, Andersson H. Women’s soccer. In AM Williams, ed, Science and Soccer: Developing Elite Performers, 3^rd^ edition. London, Routledge, 2013; p. 237-58.

Others:

1. Boshnjaku A, Dimauro I, Krasniqi E, et al. Effect of sport training on forearm bone sites in handball and soccer female players. J Sports Med Phys 2016; 56:1503-10.
2. Ebato T, Hirose N, Ono T. Availability of submaximal intermittent exercise performance test in female soccer players. Japanese J Athletic Train 2016; 2:45-50. (in Japanese)
3. Espinosa GA, Pöyhönen T, Aramendi JF, et al. Effects of an eccentric training programme on hamstring injuries in women football players. Biomed Hum Kinet 2015; 7:125-34.
4. Gilchrist J, Mandelbaum BR, Melancon H, et al. A randomized controlled trial to prevent noncontact anterior cruciate ligament injury in female collegiate soccer players. Am J Sports Med. 2008; 38:1476-83.
5. Kawamoto R, Horino H, Ota S, et al. (2015) Physical fitness profile of Japan women’s national football team for the Universide tournament. J Train Sci Exerc Sport 2015; 26:169-76. (in Japanese)
6. Oda K, Ogaki R, Murakami K, et al. Epidemiology of female collegiate soccer team injuries over three years. Rigakuryoho Kagaku 2018; 33:267-71. (in Japanese)
7. Roelofs E, Bockin A, Bosch T, et al. (2020) Body composition of National Collegiate Athletic Association (NCAA) Division 1 female soccer athletes through competitive seasons. Int J Sports Med 2020; doi: 10.1055/a-1177-0716.
8. Sakamoto K, Shimizu Y, Asai T (2014) Swing motion characteristics of instep kicks in female soccer players. Japan J Phys Educ Hlth Sport Sci 2014; 59:771-88. (in Japanese)
9. Zacher J, Blome I, Schenk A, et al. Cardiac adaptations in elite female football and volleyball athletes do not impact left ventricular global strain values: A speckle tracking echocardiography study. Int J Cardiovasc Imag 2020; 36:1085-96.
