# Supplementary figures and images for: Growth and Maturity Status of Female Soccer Players: A Narrative Review
Source: Int J Environ Res Public Health. 2021 Feb 4;18(4):1448. doi: 10.3390/ijerph18041448 (PMC7913875; doi:10.3390/ijerph18041448)

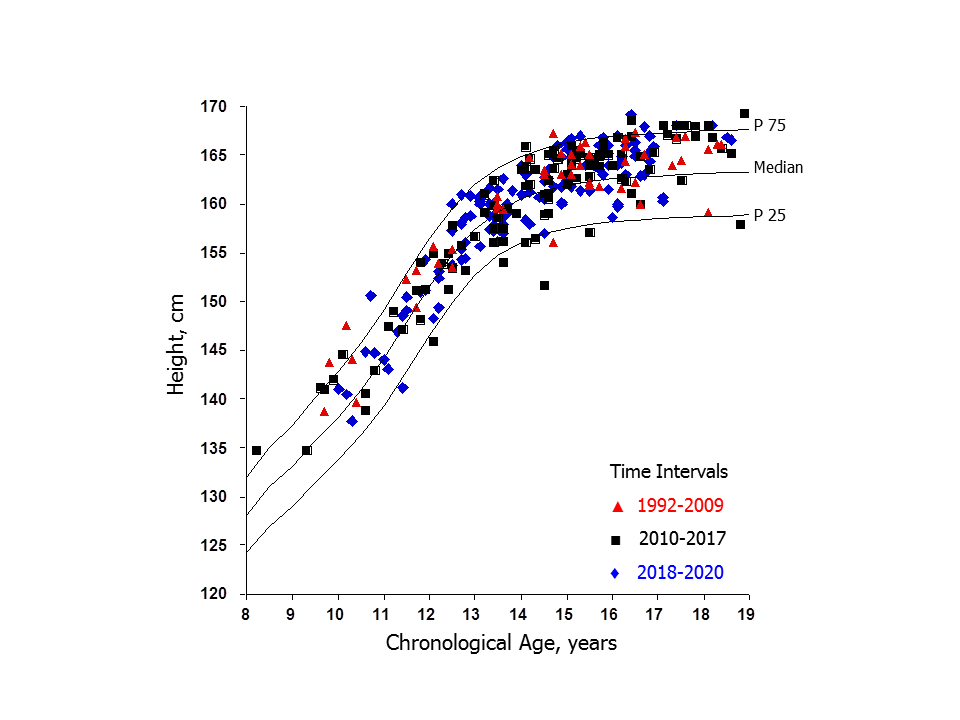

Supplement: Supplementary file 1 [file ijerph-18-01448-s001.zip › 13_Suppl-Fig1A_ht_3-intervals.tif]

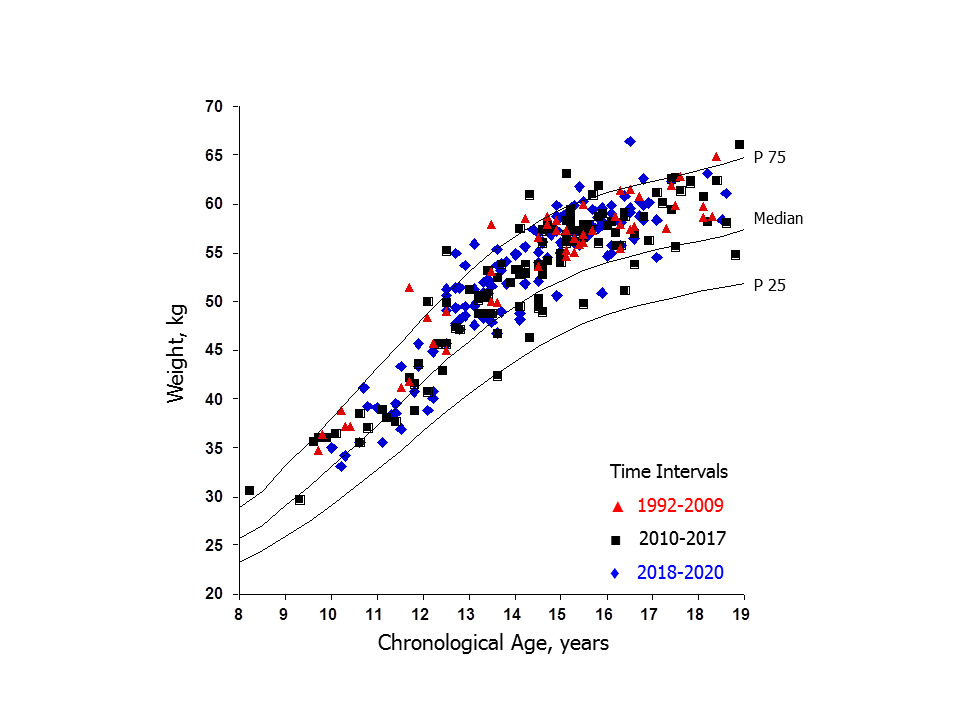

Supplement: Supplementary file 1 [file ijerph-18-01448-s001.zip › 14_Suppl-Fig1B_wt_3-intervals.tif]
